# CXCL1 and CXCL6 Are Potential Predictors for HCC Response to TACE

**DOI:** 10.3390/curroncol30030267

**Published:** 2023-03-20

**Authors:** Maximilian N. Kinzler, Katrin Bankov, Julia Bein, Claudia Döring, Falko Schulze, Henning Reis, Scherwin Mahmoudi, Vitali Koch, Leon D. Grünewald, Angelika Stehle, Dirk Walter, Fabian Finkelmeier, Stefan Zeuzem, Peter J. Wild, Thomas J. Vogl, Simon Bernatz

**Affiliations:** 1 Department of Internal Medicine I, University Hospital Frankfurt, Goethe University-Frankfurt am Main, 60590 Frankfurt am Main, Germany; 2 Dr. Senckenberg Institute for Pathology, University Hospital Frankfurt, Goethe University Frankfurt am Main, 60590 Frankfurt am Main, Germany; 3 Department of Diagnostic and Interventional Radiology, University Hospital Frankfurt, Theodor-Stern-Kai 7, 60590 Frankfurt am Main, Germany; 4 Frankfurt Cancer Institute (FCI), Goethe University, 60590 Frankfurt am Main, Germany; 5 Frankfurt Institute for Advanced Studies (FIAS), 60438 Frankfurt am Main, Germany; 6 University Cancer Center Frankfurt (UCT), University Hospital, Goethe University, 60590 Frankfurt am Main, Germany

**Keywords:** hepatocellular carcinoma, transarterial chemoembolization, immune profiling, biomarker

## Abstract

Distinct immune patterns of hepatocellular carcinoma (HCC) may have prognostic implications in the response to transarterial chemoembolization (TACE). Thus, we aimed to exploratively analyze tumor tissue of HCC patients who do or do not respond to TACE, and to identify novel prognostic biomarkers predictive of response to TACE. We retrospectively included 15 HCC patients who had three consecutive TACE between January 2019 and November 2019. Eight patients had a response while seven patients had no response to TACE. All patients had measurable disease according to mRECIST. Corresponding tumor tissue samples were processed for differential expression profiling using NanoString nCounter^®^ PanCancer immune profiling panel. Immune-related pathways were broadly upregulated in TACE responders. The top differentially regulated genes were the upregulated CXCL1 (log2fc 4.98, Benjamini–Hochberg (BH)-*p* < 0.001), CXCL6 (log2fc 4.43, BH-*p* = 0.016) and the downregulated MME (log2fc −4.33, BH-*p* 0.001). CD8/T-regs was highly increased in responders, whereas the relative number of T-regs to tumor-infiltrating lymphocytes (TIL) was highly decreased. We preliminary identified CXCL1 and CXCL6 as candidate genes that might have the potential to serve as therapeutically relevant biomarkers in HCC patients. This might pave the way to improve patient selection for TACE in HCC patients beyond expert consensus.

## 1. Introduction

Primary liver cancer is one of the leading causes of cancer death worldwide and hepatocellular carcinoma (HCC) comprises more than two thirds of cases [1,2]. The therapeutic strategy is complex and usually evaluated in interdisciplinary tumor board meetings with consensus-based decision-making [3]. Patients are frequently multimorbid, suffering from chronic liver disease with late diagnosis of HCC, limiting the potential treatment modalities. Localized disease may be treated by locoregional therapies such as embolization, microwave-, or radiofrequency ablation if the patient does not qualify for resection or transplantation [4]. Transarterial chemoembolization (TACE) is a leading option in unresectable localized HCC [4]. In conventional TACE, chemotherapeutic agents such as mitomycin C as well as an embolization agent such as ethiodized oil are injected into the tumor feeding artery +/− additional embolization agents such as microsphers [5,6]. TACE aims to induce tumor ischemia via blocking of the blood supply and enhanced antitumoral chemotherapeutic effects via retention of chemotherapeutics within the tumor environment [6]. TACE is based on the arterial hypervascularity of HCCs, which can be visualized in radiology as the imaging hallmark: arterial hyperenhancement and venous/delayed wash-out [2]. Comparative high-quality multicenter TACE trials remain scarce as the clinical practice and TACE techniques vary globally [2,6]. Of note, performing TACE in ineligible patients may cause harm and reduced overall survival [2]. Therefore, it is of utmost importance to improve the selection of patients who will most likely benefit from TACE. Efforts were made to develop clinical and laboratory scores for TACE patient selection [2,7,8,9,10,11]. However, the scores are not advised to be used outside clinical trials [2]. Consequently, the patient selection is still based on a low level of evidence, namely expert consensus.

The immunology of HCC could reveal promising targets for improved patient selection. Inflammation such as hepatitis increases the incidence of HCC following an inflammatory-based pathogenesis. The HCC immune landscape comprises a multitude of different cell types, immune cells, immune receptors, ligands, chemokines, or cytokines [12]. Tumor-associated neutrophils, myeloid-derived suppressor cells, or regulatory T-cells can modulate the tumor and immune environment with pro- or antitumorigenic effects [12]. Recent developments in immunotherapy showed promising results, prolonging survival in patients with sensitive tumors [12,13]. However, so far, studies investigating distinct immune features in primary tumor samples that predict response to TACE in HCC patients are lacking.

We hypothesized that response to TACE may depend on different HCC immune patterns. Hence, the aim of this study was to exploratively analyze HCC tissue of TACE responders and non-responders to identify potential biomarker candidates. Those candidates could pave the way to improved evidenced-based clinical decision-making in the future beyond clinical expert consensus.

## 2. Materials and Methods

### 2.1. Database

Tissue samples and patient data used in this study were provided by the University Cancer Center Frankfurt (UCT). Histopathological confirmation of HCC was assessed by expert pathologists of the Dr. Senckenberg Institute of Pathology, University Hospital Frankfurt, Frankfurt am Main, Germany. Written informed consent was obtained from all patients and the study was approved by the Institutional Review Boards of the UCT and the Ethical Committee at the University Hospital Frankfurt (project-number: SGI-10-2020). 

### 2.2. Study Design

Retrospectively, 16 HCC patients who were treated with TACE between January 2012 and November 2019 were enrolled. Inclusion criteria: (1) histologically confirmed HCC; (2) three consecutive cTACE (mitomycin C (Medac^®^, Hamburg, Germany) and Lipiodol^®^ (Guerbet GmbH, France) ± degradable starch microspheres (EmboCept^®^S, PharmaCept GmbH, Berlin, Germany); (3) all three cTACE injected in the same liver region; (4) all mRECIST target lesions (TL) treated with each cTACE; (5) post-TACE unenhanced CT one day after cTACE; (6) contrast-enhanced arterial and portal-venous/delayed phase imaging prior to the first and after the third cTACE. Exclusion criteria: (1) TLs in both liver lobes with cTACE in different liver regions; (2) time interval between first and last TACE longer than 6 months; (3) prior ablation/local therapy of TLs; (4) no measurable lesions; (5) insufficient image quality; (6) other chemotherapeutic agents than mitomycin C; (7) time interval between biopsy and treatment longer than 6 months; (8) insufficient tissue sample/RNA/NanoString quality or quantity.

### 2.3. Conventional TACE and Tumor Response Assessment

Patients were treated with cTACE in clinical routine as described in previous studies [14]. Modified response evaluation criteria in solid tumors (mRECIST) was used to assess response to TACE [15]. Complete (CR) or partial response (PR) was defined as response. Stable or progressive disease was defined as no response.

### 2.4. Ribonucleic Acid (RNA) Isolation and Immune Profiling Analysis

Pre-TACE formalin-fixed paraffin-embedded (FFPE) tissue samples were retrieved from the archive of the Dr. Senckenberg Institute of Pathology, University Hospital Frankfurt. Representative tumor material of the primary tumor was retrieved by punching out a 1 mm core. RNA was isolated using the truXTRAC FFPE total NA Kit (Covaris, Woburn, MA, USA) based on focused ultrasonication and column purification according to the manufacturer’s instructions. NanoString nCounter^®^ Platform and PanCancer immune profiling panel were used to enrich a commercially available function-specific panel of 770 genes by hybrid capture technique (NanoString, Seattle, WA, USA). The PanCancer immune profiling panel includes genes from different immune cell types (among others, B cells, T cells, NK cells); common checkpoint inhibitors; CT antigens; and genes covering both the adaptive and innate immune response. NanoString nSolver^®^ software v4 and implemented nCounter^®^ advanced analysis module v2.0.134 were used for subsequent raw data processing and normalization by internal controls following differential supervised analysis between TACE responders (*n* = 8) and non-responders (*n* = 7) according to mRECIST. Quality control was performed with default settings as previously described [16]. One sample (TACE responder) was flagged in quality control as it exceeded the threshold of the binding capacity, and we excluded this sample for further analysis. Gene expression of TACE non-responders was set as the baseline for the comparative analysis. For pathway analysis of differentially expressed genes, Enrichr [17,18] was used for functional enrichment analysis for Gene Ontology to identify gene sets for biological processes. For further differential expression analysis, we used the following cut-offs: log2 fold change ≤−2 or ≥2 and *p* < 0.05 after Benjamini–Hochberg (BH) correction. T-distributed stochastic neighbor embedding (t-SNE) analysis and plots were performed in Python 3.7.6. [19].

## 3. Results

### 3.1. Study Population

**Figure 1:**
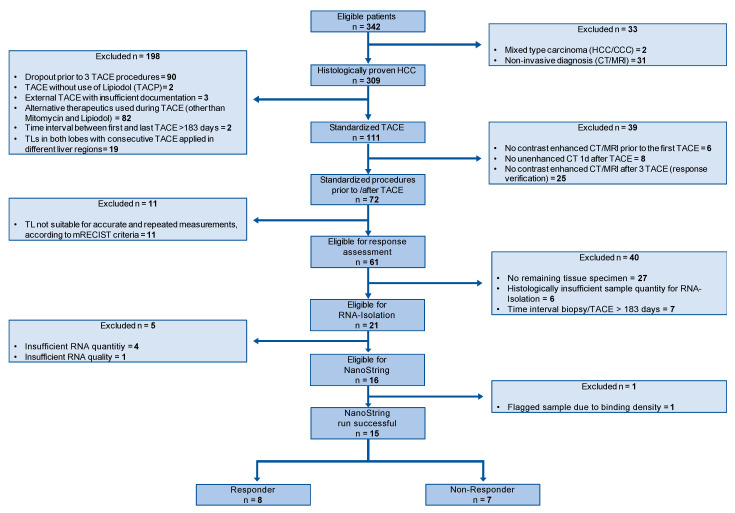
STARD flowchart of patient inclusion into the study. Abbreviations: standards for reporting diagnostic accuracy studies (STARD).

In total, 15 patients met the inclusion and exclusion criteria (Figure 1). The STARD-flowchart of patient inclusion is shown in Figure 1. Our data set comprised seven TACE non-responder (median age, 66 (54–83); men, five) and eight TACE responders (median age, 65.5 (55–75); men, seven). The groups did not differ significantly between clinical characteristics including the size of the target lesions, HCC etiology, Barcelona clinic liver cancer (BCLC) stage, Child–Pugh score, model for end-stage liver disease (MELD) score, or selected laboratory values such as alpha-fetoprotein (AFP), albumin, or bilirubin (Table 1). Further patient characteristics are shown in Table 1.

### 3.2. Unsupervised Clustering

**Figure 2:**
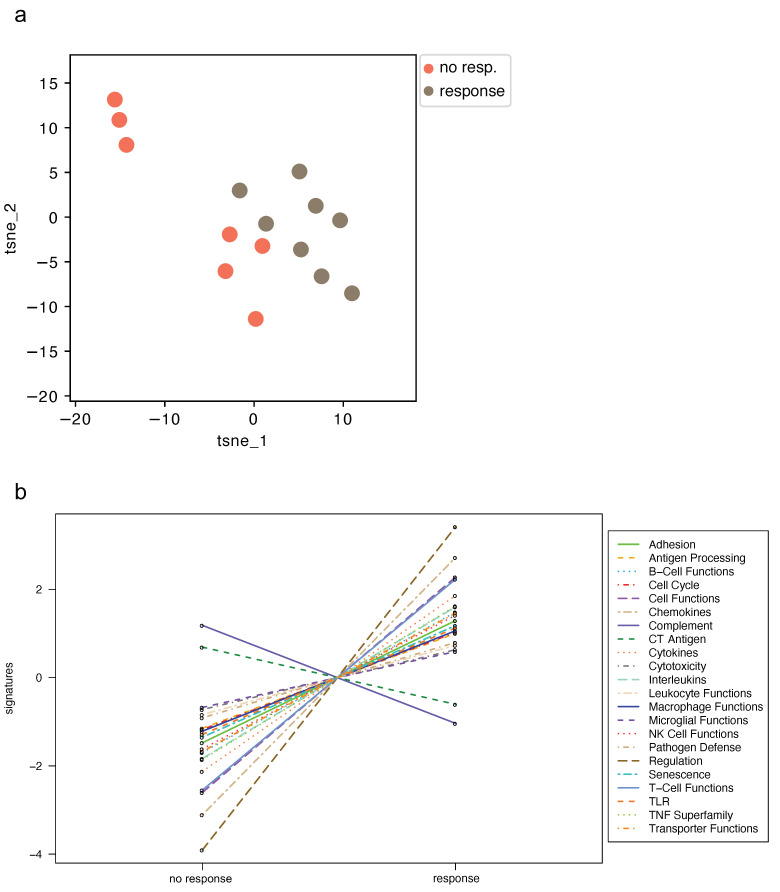
NanoString analysis reveals broad upregulation in immune pathway scores in TACE responders. (**a**) Unsupervised t-SNE plots of all normalized mRNA and (**b**) trend plot of pathway signatures. Abbreviations: messenger ribonucleic acid (mRNA), transarterial chemoembolization (TACE), T-distributed stochastic neighbor embedding (t-SNE).

Unsupervised t-SNE plots of all normalized mRNA data reveal two distinct groups of responders and non-responders (Figure 2a). NanoString pathway score analysis uses functionally annotated genes with subsequent hierarchical cluster analysis for dimensionality reduction. However, this approach did not clearly separate responders from non-responders (Figure S1). Importantly, responders reveal a general trend of upregulation in the majority of pathways; downregulation is only seen in the pathway scores describing complement and cancer/testis (CT) antigen (Figure 2b). 

### 3.3. TACE Responders Revealed Genetic Subgroup Signatures

**Figure 3:**
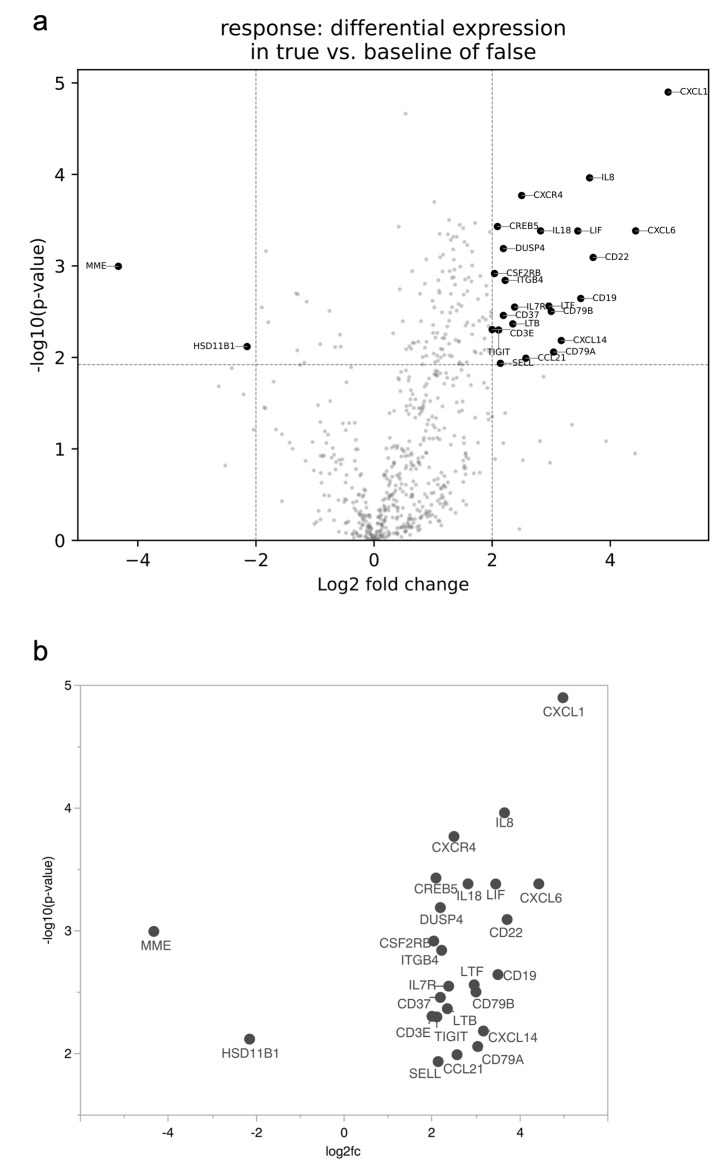
Differentially expressed genes between responders and non-responders to TACE. (**a**) Volcano plot of all differentially expressed genes or (**b**) only strong and significantly deregulated genes (log2fc ≤ −2 or ≥2 and *p*-value < 0.05 with Benjamini–Hochberg correction). Volcano plot displaying each gene’s -log10(*p*-value) and log2 fold-change with the selected covariate. Statistically significant genes fall at the top of the plot above the horizontal line, and highly differentially expressed genes fall to either side. The horizontal line indicates BH-*p* < 0.05. Abbreviations: transarterial chemoembolization (TACE).

All patients are included for preliminary expression analysis in dependence of TACE response status and are depicted in volcano plots for further analyses and subsequent selection for further functional classification. TACE responders differ in immune response-related genes (Figure 3a). A total of 25 genes are strictly deregulated (log2 fold-change ≤ −2 or ≥2, BH-*p* < 0.05) in TACE responders (Figure 3b). A total of 92% (23/25) of significantly deregulated genes were up-regulated in TACE responders and only two genes (8%, 2/25) were down-regulated (Table 2). The top three deregulated genes are the upregulated CXCL1 (log2fc 4.98, BH-*p* < 0.001) and CXCL6 (log2fc 4.43, BH-*p* = 0.016), as well as the downregulated MME (log2fc −4.33, BH-*p* = 0.001). The majority of genes (28% (7/25)) are associated with chemokines and all of them are upregulated in responders.

### 3.4. Gene Ontology Term Enrichment Analysis and Cell Type Profiling

**Figure 4:**
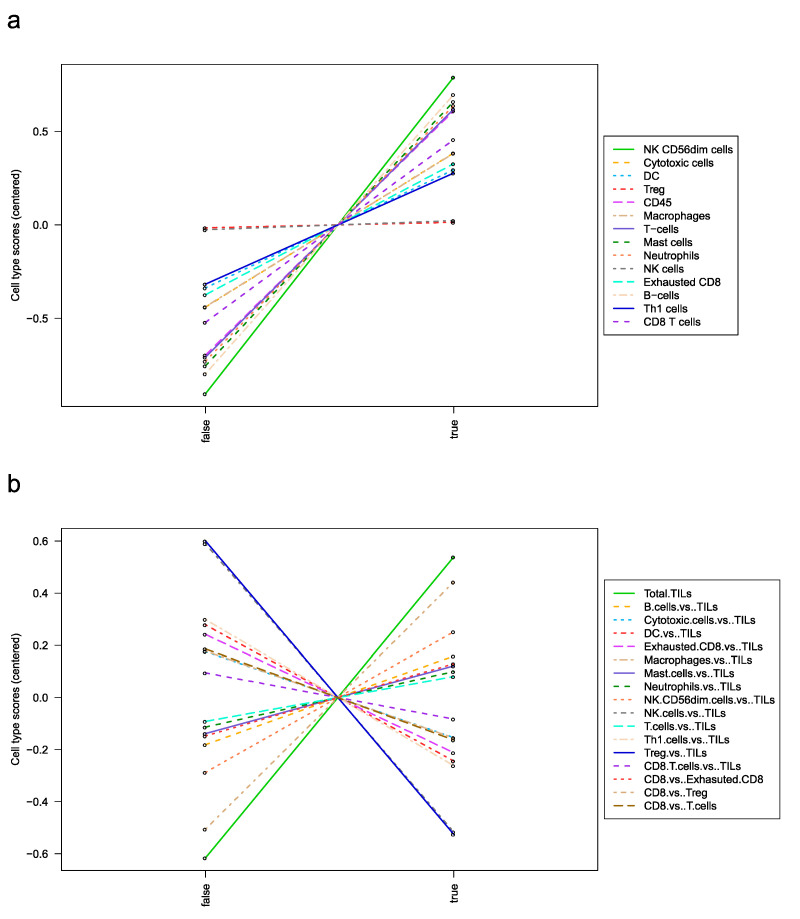
Cell type profiling reveals increased immune infiltrates in responders. (**a**) Absolute and (**b**) relative cell type scores are shown in responders (true) and non-responders (false). The nCounter^®^ advanced analysis module of the PanCancer immune profiling panel uses genes whose expression is largely specific to certain immune cell populations to measure the abundance of these cell types. The module assumes that each cell type’s characteristic genes are expressed exclusively and consistently within the cell type. Under this model, a cell type’s abundance can be measured as the average log-scale expression of its characteristic genes. The cell type abundance measurements are plotted against response. True = responder, false = non-responder.

Using Enrichr gene ontology term enrichment on the whole differentially expressed data, the biological processes were mostly related to cytokine–cytokine receptor interaction of chemokines (CC subfamily, CXC subfamily), the class I helical cytokines (mainly y-chain utilizing), and TNF family (Figure S2), as well as chemokine signaling pathways involving JAK 2/3, Src, PI3K, Itk, and PKC (Figure S3). Next, the nCounter^®^ advanced analysis module was used to measure the abundance of cell types whose expression is largely specific to certain immune cell populations [20,21]. The vast majority of immune infiltrates are increased in TACE responders (Figure 4a). The total number of tumor-infiltrating lymphocytes is increased in TACE responders. Of note, the relative number of CD8-cells to T-regs is highly increased in responders whereas the relative number of T-regs to TILs is highly decreased (Figure 4b).

## 4. Discussion

In this study, we used NanoString technology to exploratively analyze different immune patterns of HCC patients who were classified as responder and non-responder to repetitive TACE. Patients’ response to TACE was evaluated using mRECIST response assessment. We used pre-TACE tissue samples of the primary tumor and performed a multitude of NanoString technology analyses such as pathway scoring, gene set enrichment analysis, differential expression analysis, and cell type profiling to build a holistic model of different HCC immune signatures that might be associated with response to TACE. We revealed that the immune pathway scores in TACE responders are upregulated in all but two pathways. A total of 92% of the genes with the highest deregulation are upregulated in responders and the top deregulated genes are the upregulated CXCL1 and CXCL6, as well as the downregulated MME. The genes are strongly associated with the biological processes of cytokine–cytokine receptor interactions and chemokine signaling. The pathway alterations are corroborated by increased scores of tumor-infiltrating lymphocytes in responders with high CD8/T-reg and low Treg/TIL ratios.

HCC commonly arises in the background of cirrhotic liver, often as a consequence of chronic inflammation caused by viral hepatitis, alcohol abuse, metabolic disease, or exposure to a variety of substances [12]. Inflammation, as a crucial step in HCC development, is not only corroborated by reduced HCC incidence in people who are vaccinated against viral hepatitis, but also in the increased application of systemic immuno-oncological therapy [12,22]. Of note, several trials and therapeutic approaches combine immune therapy with locoregional therapies [22]. The high immunological activity in the liver is reflected in CD8 T-cells, which are major targets in immune therapy. It is known that locoregional therapies such as TACE lead to the release of tumor-associated antigens (TAAs). TAAs play a crucial role in the activation pathway of CD8 cells and their anti-tumoral activity [22]. This is in line with our results, as TACE responders reveal increased levels of CD8+ T-cells relative to T-regs. Furthermore, the number of TILs is known to reduce the rate of tumor recurrence and to prolong survival [22,23]. We also reveal an increased total number of TILs in TACE responders. Not only antitumoral cells can be found infiltrating the liver or cancerous tissue, but also immunosuppressive and, therefore, tumor-promoting cell types. Tregs lead to an immunosuppressed milieu, promoting tumor progression [22]. In our study, we reveal a strong decrease in Tregs/TILs ratio in responders, which is in line with the immuno-oncological concept. Our results suggest that pretherapeutic immune patterns may influence the response to TACE. This is of additional importance, taking into account the fact that ablative locoregional therapy can increase the spread of tumor antigens for oncolytic treatment and improve response in combination with immunotherapy [22]. Furthermore, our results show that not only response to immunotherapy but also response to the locoregional therapy itself seems to be influenced by different immune signatures in HCC. In this study, TACE responders have elevated levels of numerous members of the CC- and CXC- subfamily of chemokines, with the most prominent levels of CXCL1 and CXCL6. CXCL1 is known to promote neoplastic transformation, tumorigenesis, and angiogenesis in multiple cancer types by binding CXCR2 (CXCR2 was also increased in our analysis, data not shown). In addition, CXCL1 was described to be secreted to promote immune cell recruitment and to alter the immune milieu in liver cancer [24]. CXCL1 was further described as a factor of HCC aggressiveness, promoting proliferation and invasion, while our results indicate that CXCL1 overexpression may increase TACE response. High CXCL6 expression is described as being associated with angiogenic effects and poor prognosis in HCC [25]. CXCL6 is upregulated in TACE responders, potentially as an effect of its angiogenic ability, increasing the effect of chemoembolization. MME is a known factor in cancer cell–cell signaling and it was described to be altered by comparing pre-neoplastic tissue vs. HCC [26]. In our study, the chemokine signaling leads to the activation of JAK2/3 and PI3K. JAK and PI3K are part of signaling pathways contributing to HCC development and progression, while JAK2 inhibition has been shown to induce growth arrest in HCC cell lines [27]. However, our study has several limitations that warrant discussion. We performed a retrospective analysis and selection bias cannot be ruled out. With 15 patients (8 responders, 7 non-responders), our study population is very small, and generalizability may not be presumed. However, we followed a rigorous study design of patient inclusion and exclusion. Next, in the vast majority of cases in this setting, tissue is only available from biopsies, which strongly hinders sufficient RNA isolation due to the sample size. Thus, unstandardized interventional procedures and the limited availability of high-quality tissue specimens did not allow for a higher number of samples in our explorative study. Furthermore, this strongly limits the opportunity to perform additional immunohistochemistry as verification on protein level. Given these considerations, and the fact that data on this topic are lacking so far, the results of the present study are of high clinical interest. Our aim was to exploratively analyze different HCC immune patterns using NanoString technology. However, due to the retrospective nature of this study and limited tissue availability, confirmatory analysis such as qPCR, in vitro, and in vivo experiments should be addressed in further prospective studies.

## 5. Conclusions

In summary, HCC immune signatures may reveal biomarkers for TACE response prognostication in HCC patients. Chemokines, and especially the CC and XCX subfamily with CXCL1 and CXCL6 as two leading representatives, seem to be promising response biomarkers. Our preliminary study sheds light on the treatment-influencing relevance of the HCC immunology for TACE response beyond systemic therapy. However, our results should not be overstated, as they were derived following an explorative approach and they need to be corroborated in further multicenter studies.

## Figures and Tables

**Table 1:** Clinical and epidemiological characteristics.

	Non-Responder (*n* = 7)	Responder (*n* = 8)	*p*-Value
Sex, male	5 (71.43)	7 (87.50)	0.436
Age at TACE (years, median)	66 (54–83)	65.5 (55–75)	0.638
Size, dominant target lesion (cm, median)	5.3 (1.7–8.6)	4.05 (1.4–7.3)	0.156
HCC etiology			0.614
Hepatitis B	2 (28.57)	1 (12.50)	
Hepatitis C	3 (42.86)	4 (50.00)	
ASH/NASH	2 (28.57)	2 (25.00)	
Cryptogen	0 (0.00)	1 (12.50)	
BCLC stage			0.580
A	1 (14.29)	3 (37.50)	
B	5 (71.43)	4 (50.00)	
C	1 (14.29)	1 (12.50)	
Child–Pugh Score			0.635
A	6 (85.7)	6 (75.00)	
B	1 (14.3)	2 (25.00)	
C	0 (0.00)	0 (0.00)	
MELD Score			0.926
<6	0 (0.00)	0 (0.00)	
<10	6 (85.7)	7 (87.5)	
<15	1 (14.3)	1 (12.5)	
<20	0 (0.00)	0 (0.00)	
Albumin (g/dL)	3.8 (2.3–7.2)	3.8 (1.8–4.2)	0.401
Bilirubin (mg/dL)	0.8 (0.3–1.4)	0.95 (0.5–1.2)	0.723
INR	1.11 (1.03–1.71)	1.12 (1–1.2)	0.503
CRP (mg/dL)	0.27 (0.03–1.07)	0.34 (0.22–4.39)	0.174
AFP (ng/mL)	61.9 (3.5–3500)	30.9 (5.1–9276)	0.587

For statistical analysis, two-sided Students *t*-test was used for continuous variables and likelihood ratio for nominal/ordinal data. Data are shown as absolute numbers (%) or median (min–max). Abbreviations: alpha-fetoprotein (AFP), alcoholic steatohepatitis (ASH), Barcelona clinic liver cancer (BCLC), C-reactive protein (CRP), hepatocellular carcinoma (HCC), international normalized ratio (INR), model for end-stage liver disease (MELD), non-alcoholic steatohepatitis (NASH), transarterial chemoembolization (TACE).

**Table 2:** The strongest differentially expressed genes among TACE responders and non-responders.

	Log2fc	*p*-Value	BH *p*-Value	GENE Sets
CXCL1	4.98	<0.001	0.007	Chemokines. Regulation
CXCL6	4.43	<0.001	0.016	Chemokines. Regulation
CD22	3.71	<0.001	0.017	
IL8	3.65	<0.001	0.016	Chemokines. Cytokines. Interleukins. Pathogen defense. Regulation
CD19	3.50	0.002	0.023	B-cell functions. Regulation
LIF	3.45	<0.001	0.016	Cell functions
CXCL14	3.17	0.007	0.038	Chemokines
CD79A	3.04	0.009	0.042	
CD79B	3.00	0.003	0.026	B-cell functions
LTF	2.96	0.003	0.025	
IL18	2.82	<0.001	0.016	Interleukins. NK cell functions. T-cell functions
CCL21	2.57	0.010	0.045	Chemokines. Regulation
CXCR4	2.5	<0.001	0.016	Cell cycle. Cell functions. Chemokines. Regulation
IL7R	2.38	0.003	0.025	Cytokines
LTB	2.35	0.004	0.030	Cytokines. TNF superfamily
ITGB4	2.22	0.001	0.020	Adhesion
CD37	2.19	0.003	0.028	
DUSP4	2.19	<0.001	0.017	
SELL	2.14	0.012	0.050	Regulation
TIGIT	2.11	0.005	0.033	T-cell functions
CREB5	2.09	<0.001	0.016	
CSF2RB	2.04	0.001	0.019	Chemokines
CD3E	2.00	0.005	0.033	B-cell functions. Cell functions. T-cell functions
HSD11B1	−2.15	0.008	0.040	Cell functions
MME	−4.33	0.001	0.019	Cell functions

‘Estimated log fold-change’ estimates a gene’s differential expression. For categorical covariates, a gene is estimated to have 2^(log fold-change) times its expression in TACE non-responder baseline samples, holding all other variables in the analysis constant. The log2 and linear fold-change is also presented, along with a *p*-value and an adjusted *p*-value or FDR (BH correction). Abbreviations: Benjamini–Hochberg (BH), false discovery rate (FDR), transarterial chemoembolization (TACE).

## Data Availability

The data are available upon reasonable request from the corresponding author.

## Supplementary Materials

The following supporting information can be downloaded at: https://www.mdpi.com/article/10.3390/curroncol30030267/s1, Figure S1: Unsupervised clustering using NanoString pathway score analysis; Figure S2: Pathview: Cytokine-Cytokine Receptor Interaction; Figure S3: Pathview: Chemokine Signaling Pathway.
