# Supplementary material for: CXCL1 and CXCL6 Are Potential Predictors for HCC Response to TACE
Source: Curr Oncol. 2023 Mar 20;30(3):3516–28. doi: 10.3390/curroncol30030267 (PMC10046993; doi:10.3390/curroncol30030267)
Supplement: Supplementary file 1 [file curroncol-30-00267-s001.zip › curroncol-2125857-supplementary.pdf]

Supplementary Figures

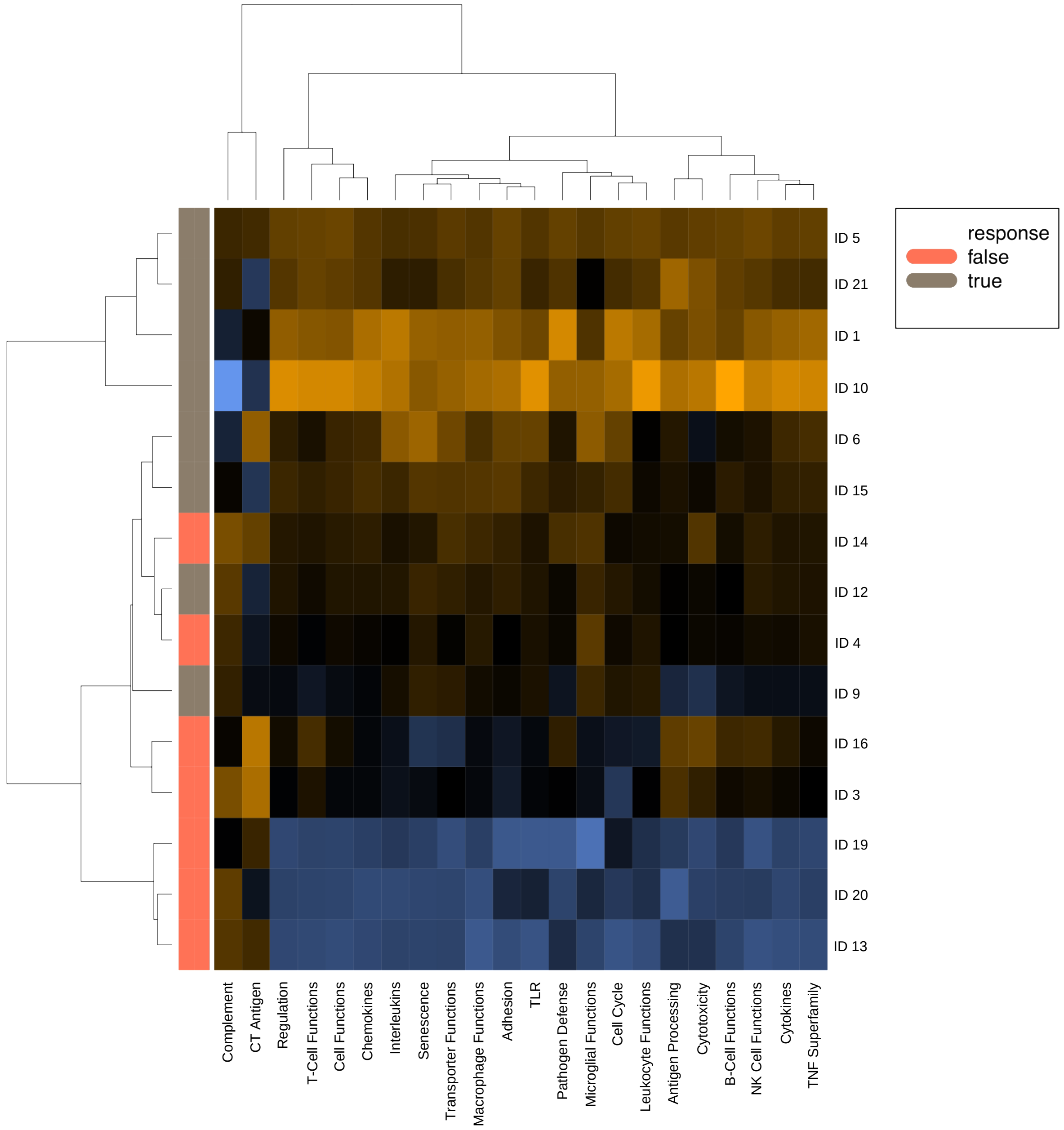

Figure. S1. Unsupervised clustering using NanoString pathway score analysis. NanoString pathway score analysis uses functionally annotated genes with subsequent hierarchical cluster analysis for dimensionality reduction. Abbreviations: Cancer/testis (CT), Natural killer cell (NK cell), Tumor necrosis factor (TNF), Toll-like receptor (TLR)

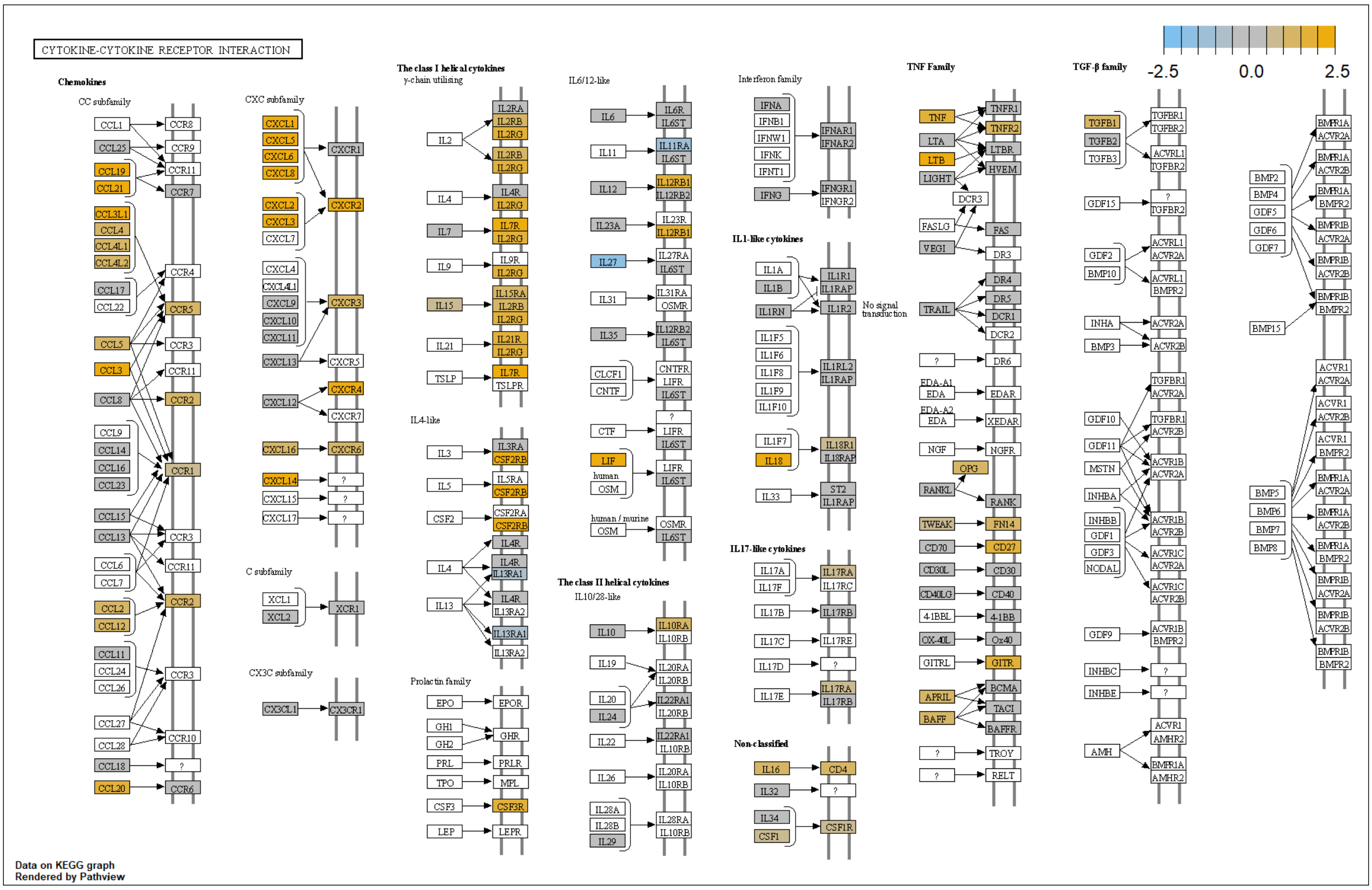

Figure. S2. Pathview: Cytokine-Cytokine Receptor Interaction. Pathview based on KEGG pathway with overlaid differential expression data. White nodes had no genes in the panel. Grey nodes were not significantly differentially expressed. Blue nodes denote downregulation and orange denote upregulation relative to our baseline of TACE non-responders. Abbreviations: Kyoto Encyclopedia of Genes and Genomes (KEGG), Transarterial chemoembolization (TACE)

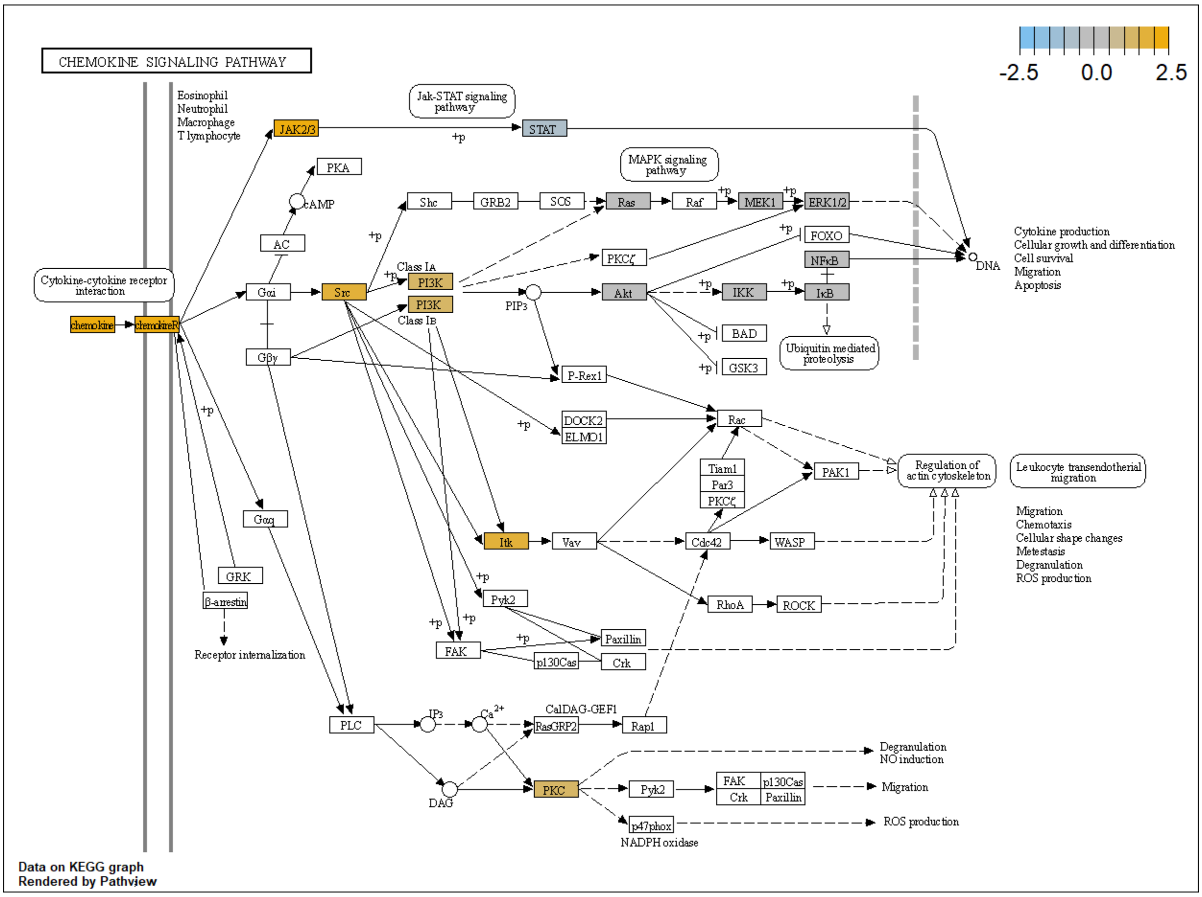

Figure. S3. Pathview: Chemokine Signaling Pathway. Pathview based on KEGG pathway with overlaid differential expression data. White nodes had no genes in the panel. Grey nodes were not significantly differentially expressed. Blue nodes denote downregulation and orange denote upregulation relative to our baseline of TACE non-responders. Abbreviations: Kyoto Encyclopedia of Genes and Genomes (KEGG), Transarterial chemoembolization (TACE).
